# Supporting research readiness in social enterprise health services

**DOI:** 10.1186/s12913-017-2607-3

**Published:** 2017-09-13

**Authors:** Nat M. J. Wright, Philippa Hearty, Linda Harris, Andrew Burnell, Sue Pender, Chris Oxnard, George Charlesworth

**Affiliations:** 1 Spectrum Community Health CIC, Wakefield, UK; 2 Nottingham University, Nottingham, UK; 3 City Health Care Partnership CIC, Hull, UK; 4 Yorkshire and Humber Clinical Research Network, Yorkshire, UK

**Keywords:** Social enterprises, Applied Health Research, Research governance, Research infrastructure

## Abstract

Health-based social enterprises are spun out of the NHS, yet continue to provide NHS-funded services. With the spin-out, however, formal processes for research governance were lost. Patients have a right to take part in research, regardless of where they access healthcare. This paper discusses the barriers to social enterprises undertaking applied health research and makes recommendations to address the need for equivalence of governance processes with NHS trusts.

Social enterprises (SEs) are businesses trading for social and environmental purposes. Rather than maximising private profit for shareholders, their aim is to reinvest all surplus back into their social and environmental goals [1]. In 2002, the UK Government released a strategy emphasising the importance of strengthening the UK’s social enterprise sector, [2] and consequently, the social enterprise sector has experienced considerable growth. Recent estimates indicate there are approximately 70,000 SEs operating in the UK [3] with 8–9% of the total providing NHS funded care to the public [1, 4]. In 2011, it was estimated that approximately 10% of community health services were either in the process of completing their business cases or had already launched as SEs, and 14% of all community nursing care services are provided by SEs [5, 6].

Such a rise in the number of social enterprises has been a response to a policy drive of increasing market competition and entrepreneurship in an effort to reduce costs of health and social care service provision [7]. This drive has been witnessed globally. For example, there is evidence of an increasing role taken by social enterprises in administering the Australian National Disability Insurance Scheme in an effort to control fiscal spending through the welfare budget [8]. However, the notion of what constitutes a social enterprise is contested, particularly in relation to organisational form. At one end of the spectrum are mutuals, akin to the philanthropic activity and cooperative movement which first arose in the late nineteenth and early twentieth centuries [9]. At the other end of the spectrum are hybrid models, whereby commentators believe that social enterprises can exist as for-profit bodies and that, indeed, the only way to ‘scale up’ social impact is to attract investment that is seeking a blended return of social gain, plus a reasonable profit on the level of risk involved in the enterprise [10]. The UK Government has supported such a model through the creation of the Community Interest Company (CIC). The CIC offers social enterprises a bespoke legal form that reconciles the inherent tensions between having a business focus and providing social benefit [11].

Therefore, social enterprises can be seen as businesses that tackle social and environmental challenges, creating jobs while prioritising impact over profit. However, where financial profits are generated, they can be shared with co-owners, staff or other social ventures [12]. So therefore, by inference, intention is key to definition, i.e. a company trading that isn’t founded, or run explicitly, to produce social benefit as a primary purpose is outside of the definition of social enterprise. This remains the case even if genuine social benefit might arise from its activity (e.g. the products of the pharmaceutical industry, or private health care providers).

Internationally, it is acknowledged that, due to their connection to civil society and the economy, models for social enterprise tend to be shaped by influences of Government, the economy, culture and wider civil society [13]. Therefore, there will be significant differences in both organisational form and function of the social enterprise - whether it be variation in non-profit and for-profit structures, the focus of their outcomes, or differences in resource mixes. Such variety can be confusing for both professionals and the lay-public. To help convey clarity we thought it would be illuminating to provide the readership with examples of well-known social enterprises, which include: *The Big Issue UK*, an organisation offering homeless people the opportunity to earn a legitimate income; Jamie Oliver’s UK restaurant *Fifteen* which provides unemployed young people with the skills, confidence, and training to become qualified chefs; and *Divine Chocolate* the first ever farmer-owned fair-trade chocolate bar, with shares owned by a co-operative of cocoa farmers in Ghana [14]. There are also examples, internationally, of social enterprises emerging in co-ordinating welfare to work schemes.

Such growth presents the potential for SEs to conduct health research. However, it is not clear whether SEs working in the health sector are eligible to access traditional funding streams for applied health research. Guidance from the UK National Institute for Health Research (NIHR) states that “for instance, programme grants and the RfPB (Research for Patient Benefit) Programme are open to NHS bodies and other providers of NHS services in England, which might include local authorities or social enterprises; other organisations can join applications for Programme Grants and RfPB as collaborators” [15]. However, researchers applying to such funding streams need to nominate a lead NHS trust which precludes SEs from being the host organisation [16].

Also, SEs are not specifically mentioned in the AcoRD guidance in which the target audience is the chief executives of NHS organisations [17]. The guidance provides a framework to identify, attribute and recover the various costs associated with research in the NHS. It applies to NHS research covered by the *Research Governance Framework for Health and Social Care* [18]. However, this framework was published ten years ago, prior to the formation of many SEs, and therefore, the social enterprise agenda is not explicit. The framework documents the responsibilities of both the sponsor organisation and the chief investigator of the research study. These responsibilities equally apply to SEs conducting research amongst its patient populations. Therefore, SEs have a responsibility to evidence their “research readiness”, by demonstrating compliance with existing research governance standards, thus providing assurance regarding SEs’ capacity and capability to recruit into research studies. Specifically, SEs are not, at this time, NIHR partner organisations and, as non-NHS bodies, they fall outside of the remit of the Health Research Authority centralised governance process. From April 2016, this process became fully operational and is the route for granting approvals for all project-based research in the NHS in England [19].

Therefore, there is a pressing need for social enterprises to develop robust governance systems that are equivalent to the standards offered by the HRA approvals process. They would need to ensure their employees have access to, and are supported to fulfil the necessary training to confer research competence. Service level agreements with Clinical Research Networks, subject to scrutiny through the Care Quality Commission (CQC) inspection process, would ensure that SEs are subject to the same accountability procedure as NHS trusts. For SEs able to demonstrate such readiness, we would argue there is a requirement for NIHR research funding panels to permit such SEs (delivering NHS funded healthcare) to be considered viable lead organisations. The SE would need to demonstrate adherence to key NIHR objectives regarding increasing the number of participants recruited into studies, whilst reducing the time to recruit first participant once permissions have been granted [20].

In summary, some NHS-funded health care services are being provided by SEs, with a risk that without an inclusive research framework, a significant proportion of the population accessing such services will be denied access to NIHR funded research activity. Clear governance frameworks would provide assurances to NIHR funding bodies from SEs seeking to lead on NIHR funded research for the benefit of their patient populations. Whilst, internationally, there are many examples of vibrant social enterprises, we were unable to retrieve an evidence base pertaining to viable models of research governance in health-based social enterprises. Therefore, in the absence of such an evidence base we would suggest there could be a variety of models worthy of implementation and subsequent evaluation. At one end of the spectrum, a social enterprise could work in partnership with an NHS trust, which would assume research governance functions for the enterprise. Alternatively, however, smaller social enterprises could form research alliances to share governance expertise. Furthermore, larger enterprises could ‘stand-alone’ in providing research governance functions of a standard outlined in the new UK Health Research Authority frameworks for research governance.

Implementation and evaluation of such models will both fulfil the responsibility to the patient populations served by social enterprises regarding their rights to take part in applied health research, and generate an evidence base which can further refine existing models for research and wider patient care.
